# Protective Effect of Irsogladine against Aspirin-Induced Mucosal Injury in Human Induced Pluripotent Stem Cell-Derived Small Intestine

**DOI:** 10.3390/medicina59010092

**Published:** 2022-12-31

**Authors:** Takuya Kanno, Takahito Katano, Isamu Ogawa, Takahiro Iwao, Tamihide Matsunaga, Hiromi Kataoka

**Affiliations:** 1 Department of Gastroenterology and Metabolism, Nagoya City University Graduate School of Medical Sciences, 1 Kawasumi, Mizuho-cho, Mizuho-ku, Nagoya 467-8601, Japan; 2 Department of Clinical Pharmacy, Graduate School of Pharmaceutical Sciences, Nagoya City University, 3-1 Tanabe-dori, Mizuho-ku, Nagoya 467-8603, Japan

**Keywords:** acetylsalicylic acid, goblet cell, human induced pluripotent stem cell, intestinal injury, permeability, small intestine

## Abstract

*Background and Objectives*: Acetylsalicylic acid (ASA) is widely used for preventing cerebrovascular and cardiovascular diseases. Gastrointestinal (GI) tract injury is one of the major complications of aspirin use, potentially leading to severe GI bleeding. However, no drugs for preventing aspirin-induced small intestinal injury have been developed. The aim of this study was to establish a human experimental model for investigating aspirin-induced small intestinal mucosal injury. In addition, we evaluated the protective effect of Irsogladine against aspirin-induced small intestinal mucosal injury using human induced pluripotent stem cell-derived 2D monolayer crypt-villus structural small intestine (2D-hiPSC-SI). *Materials and Methods*: Human iPS cell-derived intestinal organoids were seeded and cultured in Air-liquid interface. The permeability of 2D-hiPSC-SI was evaluated using Lucifer yellow. Changes in structure and mucosal permeability of 2D-hiPSC-SI after addition of aspirin were confirmed over time, and changes in intestinal epithelium-related markers were evaluated by real-time qPCR and Immunofluorescence staining. The effect of Irsogladine on prevention of aspirin mucosal injury was examined by adding Irsogladine to the culture medium. *Results*: Cultured 2D-hiPSC-SI showed multi-lineage differentiation into small intestinal epithelium comprised of absorptive cells, goblet cells, enteroendocrine cells, and Paneth cells, which express CD10, MUC2, chromogranin A, and lysozyme, respectively. RNA in situ hybridization revealed intestinal stem cells that express *Lgr5*. ASA administration induced an increase in the mucosal permeability of 2D-hiPSC-SI. ASA-injured 2D-hiPSC-SI showed decreased mRNA expression of multi-lineage small intestinal cell markers as well as intestinal stem cell marker *Lgr5*. Administration of Irsogladine on the basal side of the 2D-hiPSC-SI resulted in significant increases in *Mki67* and *Muc2* mRNA expression by 2D-hiPSCs at 48 h compared with the control group. Administration of 400 µg/mL Irsogladine to the ASA-induced small intestinal injury model resulting in significantly decreased mucosal permeability of 2D-hiPSC-SI. In immunofluorescence staining, Irsogladine significantly increased the fluorescence intensity of MUC2 under normal conditions and administration of 400 µg/mL ASA. *Conclusions*: we established a novel ASA-induced small intestinal injury model using human iPSC-derived small intestine. Irsogladine maintains mucosal permeability and goblet cell differentiation against ASA-induced small intestinal injury.

## 1. Introduction

Acetylsalicylic acid (ASA), also known as aspirin, is used as an anti-inflammatory drug. Low-dose-aspirin (LDA), defined as less than 325 mg per day, prevents cerebrovascular and cardiovascular diseases and is therefore widely used worldwide [1,2,3]. LDA exerts antithrombotic effects by suppressing cyclooxygenase-1 activity, which leads to decreased prostaglandin (PG) production. As PGs play an important role in maintenance of the gastrointestinal (GI) epithelium by upregulating mucosal blood flow, a decrease in PG synthesis can cause GI mucosal damage [4]. Hence, GI tract injury is a major adverse event of LDA, potentially leading to severe GI bleeding [2,5]. The mortality rate from gastrointestinal bleeding in the upper and lower GI tract is approximately 10% and 3%, respectively [6,7]. In particular, the mortality rate within 1 year in heart failure patients who experienced bleeding events is reportedly >50% [8]. Thus, prevention of LDA-induced GI injury is an important issue in clinical practice.

Vonoprazan and proton pump inhibitors (PPIs) reduce the incidence of LDA-induced gastric and duodenal ulcers, and therefore, these drugs are recommended for preventing upper GI injury related to LDA [9,10,11]. However, no drugs are approved for the prevention of drug-induced (including LDA-induced) small intestinal injury, which accounts for 41% of cases of small intestinal bleeding [12,13]. One randomized controlled trial (RCT) reported that high-dose rebamipide, a gastroprotective drug, might be effective for the treatment of LDA-induced moderate-to-severe small intestinal injury. However, the dose of rebamipide used in the RCT was triple the dose approved for medical care [14]. Therefore, the results of that study do not repudiate the efficacy of standard-dose rebamipide, and the need remains for a drug to prevent LDA-induced small intestinal injury. Another previous RCT showed that Irsogladine (2,4-diamino-6-[2,5-dichlorophenyl]-s-triazine), a gastroprotective drug widely used in Japan, inhibits small intestinal mucosal injury induced by diclofenac sodium, which is categorized as a non-steroidal anti-inflammatory drug (NSAID) [15]. Hence, Irsogladine is a candidate drug for preventing LDA-induced small intestinal injury.

Mouse models have been developed as research tools to reproduce NSAID- and LDA-induced small intestinal mucosal injury [16]. However, the immune response, microbiota, and pharmacokinetics of mice differ from humans. Therefore, animal models cannot reconstitute the environment of the human digestive system [17,18]. Caco-2 human colorectal cancer cells are also used for small intestinal research. However, the drug-metabolizing enzyme activity and tight junction functions of these cells differ significantly from those of healthy human small intestine [19]. Thanks to recent advances in tissue engineering, intestinal organoid culture systems have been developed as useful tools for intestinal stem cell (ISC) research and drug screening [20,21]. Small intestinal organoids cultured in Matrigel exhibit a three-dimensional (3D) cystic structure with budding formations and maintain similar in vivo ISC functions over the long term. Organoids can be generated from human induced pluripotent stem cells (iPSCs), and we developed human iPSC-derived small intestinal organoids [22,23,24] that express not only various differentiated epithelial cell markers but also pharmacokinetic-related genes, such as *CYP3A4* and *ABCB1/MDR1* [24]. Therefore, human iPSC-derived small intestine could be an ideal research tool for drug-induced injury or drug screening studies. However, organoids have certain drawbacks associated with the 3D structure. The apical side of the intestinal epithelium is inside the cultured organoid, which impedes direct access of external factors to the intestinal inner lumen. Hence, 3D-organoids are not suitable research tools for drug absorption and transport studies. To overcome this drawback, we recently established a two-dimensional (2D) monolayer culture system from human iPSC-derived intestinal organoids [25]. Cells seeded in a cell culture insert from iPSC-derived intestinal organoids formed a villus structure toward the upper air-side, which improved the versatility for experimental manipulation. Thus, the 2D monolayer culture system is an ideal alternative tool for evaluating drug-induced epithelial injury, epithelial barrier function, and permeability.

Here, we report a novel ASA-induced small intestinal epithelial injury model using human iPSC-derived monolayer crypt-villus structural small intestine (2D-hiPSC-SI) cultured at the air-liquid interface (ALI). Using this model, we also show that Irsogladine prevents ASA-induced small intestinal damage.

## 2. Materials and Methods

### 2.1. 2D-hiPSC-SI

Human iPS cells (Windy) were cultured as reported previously [24,25]. A modified version of a previously described differentiation protocol for induction at day 7 was used. The cells were trypsinized for 3 min, and then filtered through a cell strainer using a 40-µm nylon-mesh. The cells were subsequently seeded onto plates coated with 0.16 µg/mL laminin-511 E8 fragment (Nippi, Tokyo, Japan) and cultured with medium containing 5% fetal bovine serum (FBS), 100 ng/mL epidermal growth factor (EGF), 3 μM CHIR99021, 500 nM A-83-01, 10 μM Y-27632, and 30 μg/mL fibroblast growth factor 2 (FGF2). After the cells reached 90% confluence, they were passaged onto laminin-511 coated plates and cultured for 72–96 h. The cells were then dissociated and seeded onto EZSPHERE plates and cultured for 3 days to generate spheroids. The spheroids were cultured in Advanced DMEM/F-12 (ADF) containing 3% Matrigel for an additional 6–18 days to generate intestinal organoids. The organoids were pre-incubated with 10 µM Y27632 for 3 h, centrifuged at 200× *g* for 5 min, washed once with phosphate-buffered saline (PBS), collected into a tube, and incubated in PBS with 0.5 mM EDTA (Thermo Fisher Scientific, Waltham, MA, USA) for 5 min at room temperature. Following a centrifugation of the organoids, a pellet was created that was suspended and incubated in TrypLETM Select (Thermo Fisher Scientific, Waltham, MA, USA) for 15 min at 37 °C. The cells were suspended in ADF containing 10% FBS, centrifuged at 200× *g* for 5 min at room temperature, and the suspension was seeded onto 24-well cell culture inserts with a pore size of 0.4 µm. The cells were cultured in ADF containing 2% FBS, 1% GlutaMAX, 15 mM HEPES, 1% N2 supplement, 2% B27 serum-free supplement, 100 U/mL penicillin, and 100 µg/mL streptomycin (conditioned medium) with additional supplements for the first 3 days, as shown in Figure S1. The cells were then cultured at the ALI, and the medium was changed every 2–3 days.

### 2.2. ASA-Induced Small Intestinal Injury Model

On the eighth day of 2D-hiPSC-SI culture, ASA (Tokyo Chemical Industry Co., Ltd., Tokyo, Japan) was dissolved in basal medium at concentrations of 400 µg/mL, 1200 µg/mL, and 2400 µg/mL and added to the basal side of the 2D-hiPSC-SI and incubated for 48 h. The aspirin concentrations were set considering the intestinal drug concentration as calculated using the following formula: clinical dose (mg)/250 mL.

### 2.3. Evaluation of the Effect of Irsogladine on ASA-Induced Small Intestinal Injury

On the seventh day of 2D-hiPSC-SI culture, Irsogladine maleate (Tokyo Chemical Industry Co., Ltd., Tokyo, Japan) was administered in basal medium at a concentration of 16 µg/mL and incubated for 48 h. On the eighth day of culture, ASA was administered in basal medium at concentrations of 400 µg/mL and 1200 µg/mL. Samples were collected 24 h after ASA administration for further analysis. The Irsogladine concentration was set considering the intestinal drug concentration as calculated using the following formula: clinical dose (mg)/250 mL.

### 2.4. RNA Extraction, Reverse Transcription, and Real-Time PCR

The mRNA expression levels of the *Lgr5*, *Mki67*, *Muc2*, *Vil1*, *Chga*, *Lyz*, *Cdh1*, and *Hprt* genes in 2D-hiPSC-SI were measured by real-time reverse transcription PCR (RT-PCR). *Hprt* was used as an endogenous control for data standardization. Total RNA was isolated from 2D-hiPSC-SI using an Agencourt RNAdvance Tissue kit (Beckman Coulter Inc., Brea, CA, USA). In order to create single-stranded cDNAs, 0.5 µg of total RNA was used. ReverTra Ace qPCR RT Master Mix (TOYOBO, Osaka, Japan) was used for the reverse transcription process in accordance with the manufacturer’s instructions. Real-time PCR analysis was carried out on an Eco Real-Time PCR system using Eco Real-Time PCR System software, version 5.0 (Illumina Inc., San Diego, CA, USA). PCR was performed with the primer pairs indicated in Table S1 using KAPA SYBR FAST qPCR Kit Master mix (2×) and an ABI Prism system (Sigma-Aldrich, St. Louis, MO, USA) [26].

### 2.5. Histology and Histochemistry

Cultured 2D-hiPSC-SI on cell culture inserts was fixed in 10% formaldehyde, coated with collagen gel, processed routinely, and embedded in paraffin. The samples were then sectioned and stained with hematoxylin and eosin (HE). As in vivo human small intestine samples, we used a formalin-fixed and paraffin-embedded terminal ileum specimen in colectomy. The study was performed in accordance with the Declaration of Helsinki, and the protocol was approved by the institutional review board of Nagoya City University Hospital.

### 2.6. Immunohistochemical Staining

Immunohistochemical (IHC) staining was performed using antibodies against the following antigens: MUC2 (1:100; Novocastra Laboratories, Newcastle, UK), CD10 (1:200; Novocastra Laboratories), chromogranin A (1:200; Ab45179, Abcam PLC, Cambridge, UK), and lysozyme (1:50; GTX72913, GeneTex Inc., Irvine, CA, USA). For immunohistochemical staining, paraffin sections on slides were deparaffinized using xylene and a graded ethanol series. After soaking in 0.3% H_2_O_2_/methanol solution to inhibit endogenous peroxidase activity, the sections were treated with the appropriate primary antibodies, carefully rinsed with PBS, and then incubated with biotinylated secondary antibodies followed by avidin-biotinylated horseradish peroxidase complex to block endogenous peroxidase activity. Immune complexes were clarified by incubation in 0.01% H_2_O_2_ and 0.05% 3,3′-diaminobenzidine tetrachloride (DAB). Nuclei were counterstained using Mayer’s hematoxylin [27].

### 2.7. Immunofluorescence Staining

Immunofluorescence staining was performed using antibodies against the following antigens: MUC2 (1:100; sc-15334, Santa Cruz Biotechnology, Dallas, TX, USA). 2D-hiPSC-SI grown on a cell culture insert was fixed with methanol, followed by blocking for 20 min in PBS containing 5% FBS. The samples were then reacted with primary antibodies at room temperature for 1 h, washed with PBS, and then incubated with secondary antibodies at room temperature for 1 h. Nuclei were counterstained using 4′,6-diamidino-2-phenylindole (DAPI). Furthermore, small intestine samples were washed and cut with the membrane. Finally, specimens were attached to glass slides and mounted using SlowFade Diamond Antifade Mountant. Confocal images were captured using a BZ-X810 microscope (KEYENCE, Osaka, Japan) [26].

### 2.8. RNA In Situ Hybridization

RNA in situ hybridization (RNA-ISH) of *Lgr5* was performed using an RNAscope FFPE assay kit (Advanced Cell Diagnostics, Inc., Hayward, CA, USA) according to the manufacturer’s instructions. Tissue slices (4-μm thick) that had been formalin-fixed and paraffin-embedded were immediately processed with heat and protease digestion. The sections were then hybridized with a target probe specific for *Lgr5*, followed by horseradish peroxidase–based amplification. DAB was added to evaluate the target RNA. Positive staining was judged visually as the presence of brown dots.

### 2.9. Lucifer Yellow Permeability

Lucifer yellow CH dilithium salt (LY) (Wako, Osaka, Japan) was dissolved in dimethyl sulfoxide to prepare a 20 mM stock solution. Next, 600 µL of medium on the basal (plate) side of the 2D-hiPSC-SI was replaced with HBSS and incubated at 37 °C for 30 min. Medium on the apical (cell culture insert) side of the 2D-hiPSC-SI was replaced with 100 µL of HBSS containing 110 µM LY, and 50 µL of HBSS (liquid A) was immediately collected and incubated at 37 °C for 30 min. After incubation, the HBSS on the basal side was stirred thoroughly, and then 50 µL (liquid B) was collected. Finally, the fluorescence of liquid A and liquid B was measured using a multi-plate reader to examine the permeability of the 2D-hiPSC-SI.

### 2.10. Statistical Analysis

Statistical analysis was carried out using GraphPad Prism software, version 9.2.0 for Windows (GraphPad Software, San Diego, CA, USA). Two-tailed Dunnett’s tests or two-tailed Student’s *t*-tests were used as appropriate. The number of samples is indicated by “*n*”. At least two independent replicates were performed for all experiments. Data are represented as the mean ± standard deviation (SD). A *p* value < 0.05 was considered statistically significant.

## 3. Results

### 3.1. 2D-hiPSC-SI Cultured at the ALI

**Figure 1:**
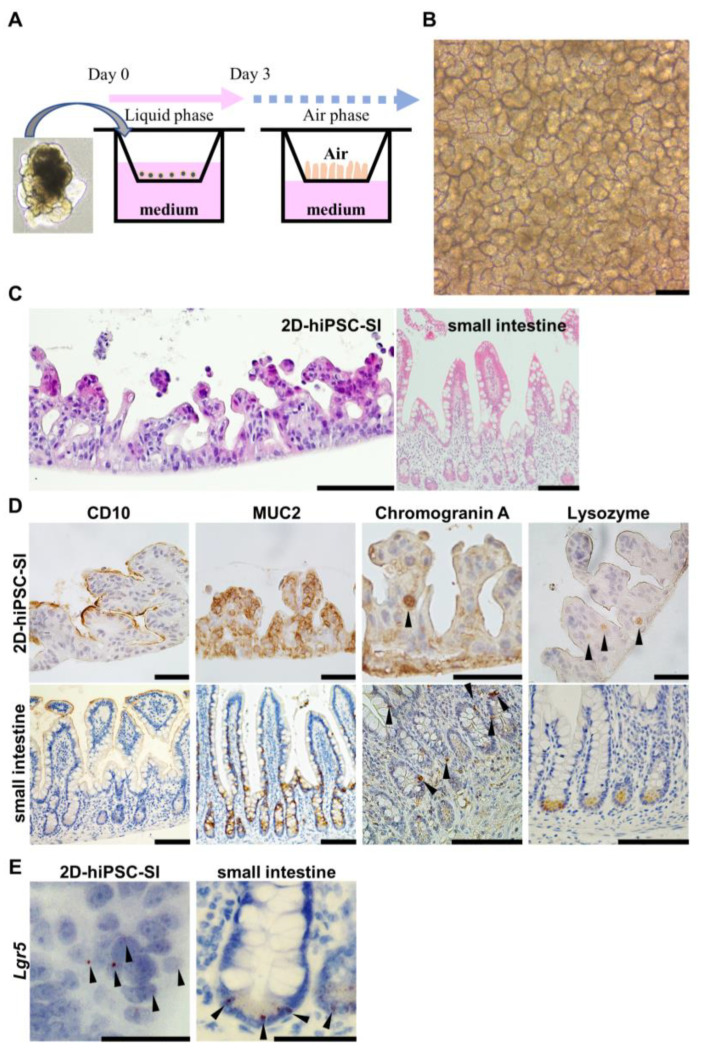
Multi-lineage differentiation of human iPSC-derived 2D monolayer small intestine. (**A**) Schematic diagrams of human iPSC-derived 2D monolayer crypt-villus structural small intestine (2D-hiPSC-SI). Cells were seeded from human iPSC-derived small intestinal budding organoids and cultured at the ALI. (**B**) Phase-contrast microscopy on culture day 8. (**C**) Hematoxylin-eosin staining showed a crypt-villus structure in cultured 2D-hiPSC-SI at day 8, similar to the small intestinal epithelium in vivo. (**D**) Immunohistochemistry staining for intestinal epithelial cell markers. CD10, absorptive cell marker; MUC2, goblet cell marker; chromogranin A, enteroendocrine cell marker; lysozyme, Paneth cell marker. Left, 2D-hiPSC; right, human small intestine in vivo. (**E**) RNA in situ hybridization of *Lgr5* of our model and human small intestine. Black arrowheads show positive staining for the respective markers. Scale bars: 100 µm (**B**–**D**); 50 µm (**E**).

We used 2D-hiPSC-SI in this study, which was established according to a modified previously reported protocol (Figure 1A and Figure S1). Cultured cells within ALI conditions exhibited a highly differentiated villus structure (Figure 1B). In HE and IHC analyses, we confirmed the crypt-villus structure of 2D-hiPSC-SI, which revealed CD10-, MUC2-, chromogranin A-, and lysozyme-positive cells and markers of absorptive cells, goblet cells, enteroendocrine cells, and Paneth cells, respectively (Figure 1C,D). RNA-ISH revealed that 2D-hiPSC-SI contained ISCs expressing *Lgr5* (Figure 1E). Thus, cultured epithelium of 2D-hiPSC-SI within ALI exhibited multi-lineage differentiation and maintenance of stem cell function, similar to in vivo human small intestine.

### 3.2. ASA-Induced Small Intestinal Injury in 2D-hiPSC-SI

**Figure 2:**
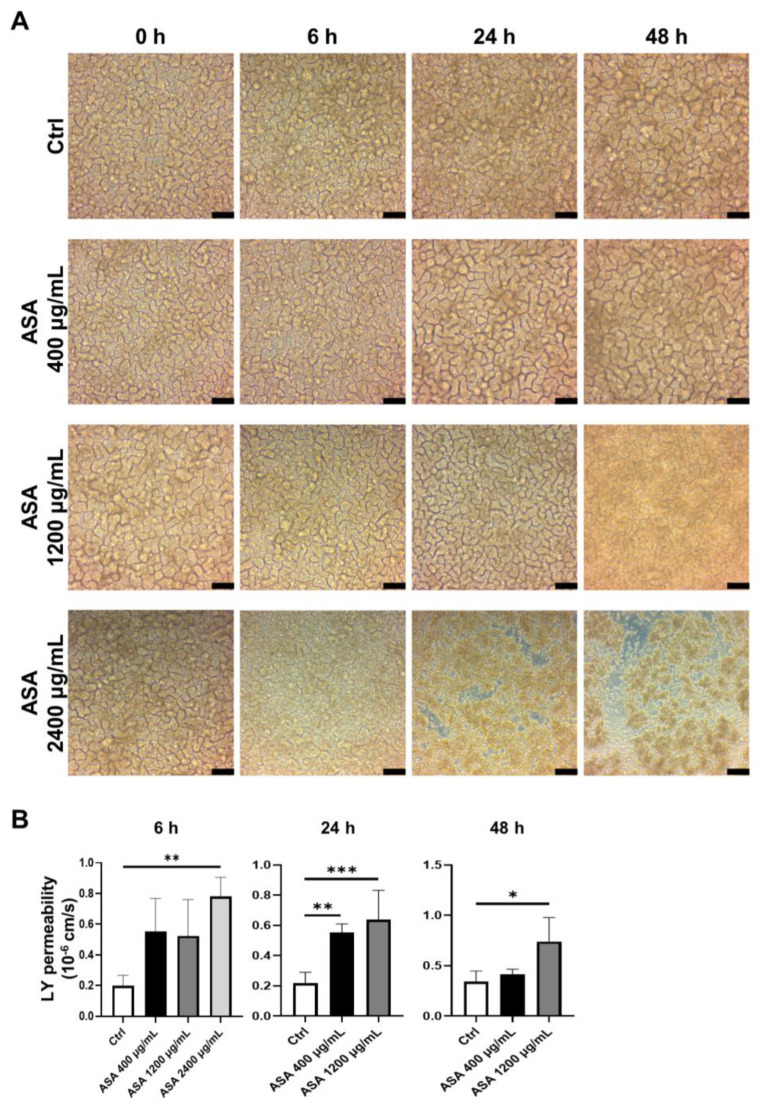
Acetylsalicylic acid (ASA) induced small intestinal injury in 2D-iPSC-SI. (**A**) Time-course analysis of cultured 2D-iPSC-SI under different ASA concentrations. The extent of damage increased in an ASA concentration-dependent manner. (**B**) Relative LY permeability indicated increased small intestinal permeability after ASA incubation. LY permeability increased significantly at 6 h in the ASA 2400 µg/mL group compared with the control group (ASA 2400 µg/mL vs. control, *p* < 0.01). At 24 h, LY permeability was significantly higher in the ASA 400 µg/mL and ASA 1200 µg/mL groups than control group (*p* < 0.01 and *p* < 0.001, respectively). At 48 h, LY permeability was significantly higher in the ASA 1200 µg/mL group than control group (*p* < 0.05); mean ± SD; n = 3–5. Statistical analysis was performed using two-tailed Dunnett’s test; * *p* < 0.05, ** *p* < 0.01, *** *p* < 0.001. ASA: acetylsalicylic acid, Ctrl: control, LY: Lucifer yellow.

To investigate of the effects of ASA on the small intestinal mucosa, ASA was added to the basal side (well-plate side) of the 2D-iPSC-SI on culture day 8, when morphological examination of the 2D-iPSC-SI revealed a mature villus structure under ALI culture conditions (Figure 2A). Time-course analysis showed that the macroscopic appearance was similar to that of the control (Figure 2A). In the ASA 400 µg/mL group, the villus height was flattened at 24 h and 48 h compared with the control group. In the ASA 1200 µg/mL group, the villus height was flattened at 24 h. In the ASA 1200 µg/mL group, the villus structure was damaged, and detachment of cultured cells was observed at 48 h. In the ASA 2400 µg/mL group, villus structural damage was observed at 6 h, and notable thinning of the villus structure along with detachment of cultured 2D-hiPSC-SI were observed between 24 and 48 h (Figure 2A).

The barrier function of the 2D-hiPSC-SI was evaluated based on LY permeability (Figure 2B). A significant increase in LY permeability was observed at 6 h in the ASA 2400 µg/mL group compared with the control group (*p* < 0.01). According to the high degree of detachment of cultured 2D-hiPSC-SI, we could not evaluate at 24 h and 48 h in the ASA 2400 µg/mL group. At 24 h, LY permeability was significantly greater in the ASA 400 µg/mL and 1200 µg/mL groups than in the control group (*p* < 0.01 and *p* < 0.001, respectively). At 48 h, LY permeability was significantly greater in the ASA 1200 µg/mL group than the control group (*p* < 0.05). Thus, ASA induced an increase in the permeability of the small intestine.

### 3.3. Changes in mRNA Expression of Intestinal Epithelial Cell Markers after ASA-Induced Small Intestinal Injury

**Figure 3:**
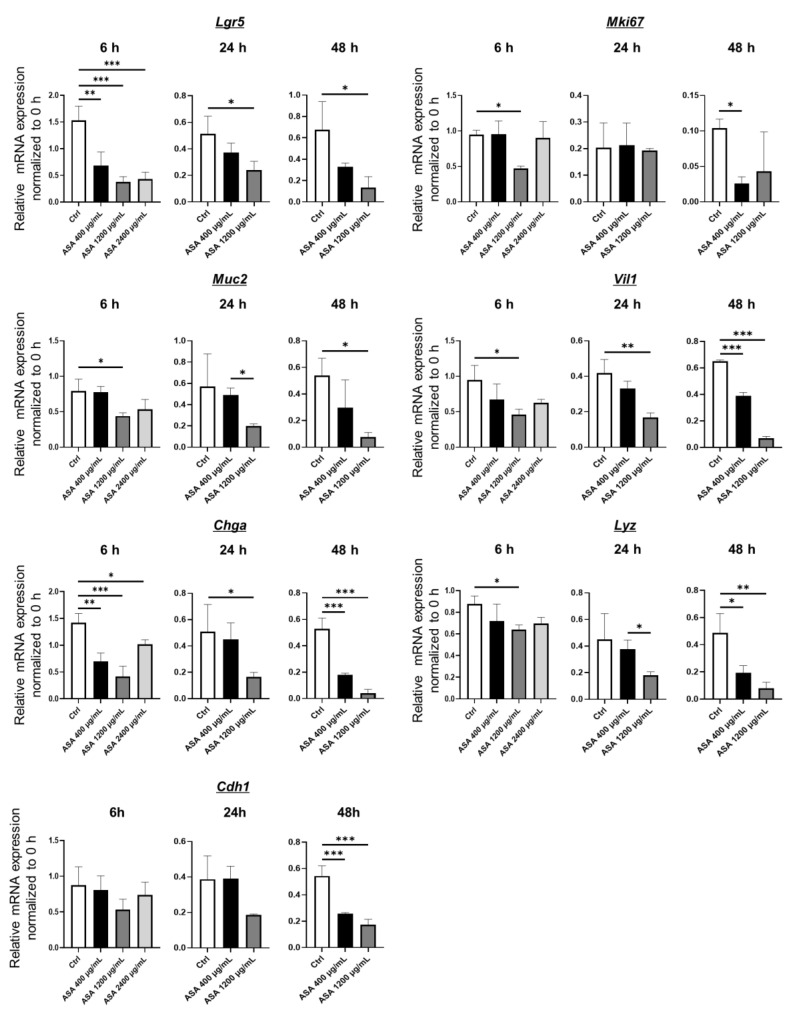
Real-time quantitative RT-PCR analysis of intestinal epithelial cell markers in ASA-injured 2D-hiPSC-SI. ASA-injured 2D-hiPSC-SI showed decreased mRNA expression of multi-lineage small intestinal cell and ISC markers. Data show mean ± SD; *n* = 3. Statistical analysis was performed using two-tailed Dunnett’s test; * *p* < 0.05, ** *p* < 0.01, *** *p* < 0.001. ASA: acetylsalicylic acid.

mRNA expression was compared in control and ASA-induced injury 2D-hiPSC-SI (Figure 3 and Figure S2). Expression of the intestinal epithelial markers Lgr5, Mki67, Muc2, Vil1, Chga, Lyz, and Cdh1 was examined using real-time quantitative RT-PCR. Expression of the ISC marker *Lgr5* in the ASA 400 µg/mL, 1200 µg/mL, and 2400 µg/mL groups was significantly decreased at 6 h compared with the control group (*p* < 0.001). At 24 and 48 h, *Lgr5* expression in the ASA 1200 µg/mL group was significantly lower than that of the control. Expression of the proliferative cell marker *Mki67* in the ASA 1200 µg/mL group at 6 h and the 400 µg/mL group at 48 h was significantly decreased compared with the control group (*p* < 0.05). Expression of the goblet cell marker *Muc2* in the ASA 1200 µg/mL was significantly decreased at 6 and 48 h compared with the control group (*p* < 0.05). Expression of the absorptive cell marker *Vil1* in the ASA 1200 µg/mL group was significantly lower than that of the control group at 6, 24, and 48 h (*p* < 0.05, *p* < 0.01, and *p* < 0.001, respectively). In the ASA 400 µg/mL group, *Vil1* expression at 48 h was also significantly lower than that of the control group (*p* < 0.001). Expression of the enteroendocrine cell marker *Chga* in the ASA 400 µg/mL, 1200 µg/mL, and 2400 µg/mL groups at 6 h was significantly lower than that of the control group (*p* < 0.01, *p* < 0.001, and *p* < 0.05, respectively). Significantly lower expression of *Chga* compared with the control was also observed in the ASA 1200 µg/mL group at 24 h and the ASA 400 µg/mL and 1200 µg/mL groups at 48 h (*p* < 0.05, *p* < 0.001, and *p* < 0.001, respectively). Expression of the Paneth cell marker *Lyz* in the ASA 1200 µg/mL group at 6 h and the ASA 400 µg/mL and 1200 µg/mL groups at 48 h was significantly decreased compared with the control group (*p* < 0.05, *p* < 0.05, and *p* < 0.01, respectively). Expression of the tight junction marker *Cdh1* in the ASA 400 µg/mL and 1200 µg/mL groups at 48 h was significantly lower than that of the control group (*p* < 0.001 each). Thus, small intestine tissue injured by ASA exhibited decreased expression of multi-lineage differentiated cell and ISC markers.

### 3.4. Effects of Irsogladine on 2D-hiPSC-SI under Normal Conditions

**Figure 4:**
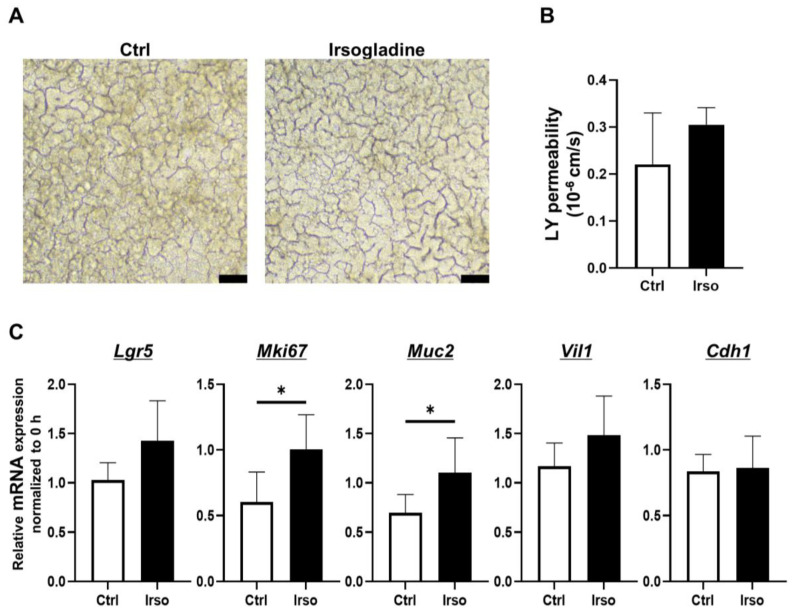
Effects of Irsogladine in 2D-hiPSC-SI under normal condition. Irsogladine was administered in culture medium for 48 h beginning on day 7. (**A**) Phase-contrast microscopy analysis on day 9. Scale bars, 100 µm. (**B**) No significant difference in LY permeability was observed between the control and Irsogladine groups (*p* = 0.4762); mean ± SD; *n* = 3. (**C**) Relative mRNA expression of *Mki67* and *Muc2* in 2D-hiPSC-SI cultured with Irsogladine for 48 h was significantly higher than that of the control group (*p* < 0.05 each); mean ± SD; *n* = 3. Statistical analysis was performed using two-tailed Student’s *t*-test; * *p* < 0.05. Ctrl: control, LY: Lucifer yellow.

To investigate the effects of Irsogladine on 2D-hiPSC-SI under normal conditions, Irsogladine was added to the basal side of 2D-hiPSC-SI and incubated for 48 h. The macroscopic appearance is shown in Figure 4A. In terms of LY permeability, there was no significant difference between the control and Irsogladine groups (Figure 4B). Real-time quantitative RT-PCR analysis revealed that expression of *Mki67* and *Muc2* was significantly increased in the Irsogladine group compared with the control group at 48 h (*p* < 0.05 each) (Figure 4C).

### 3.5. Irsogladine Plays a Protective Role against ASA-Induced Injury in 2D-hiPSC-SI

**Figure 5:**
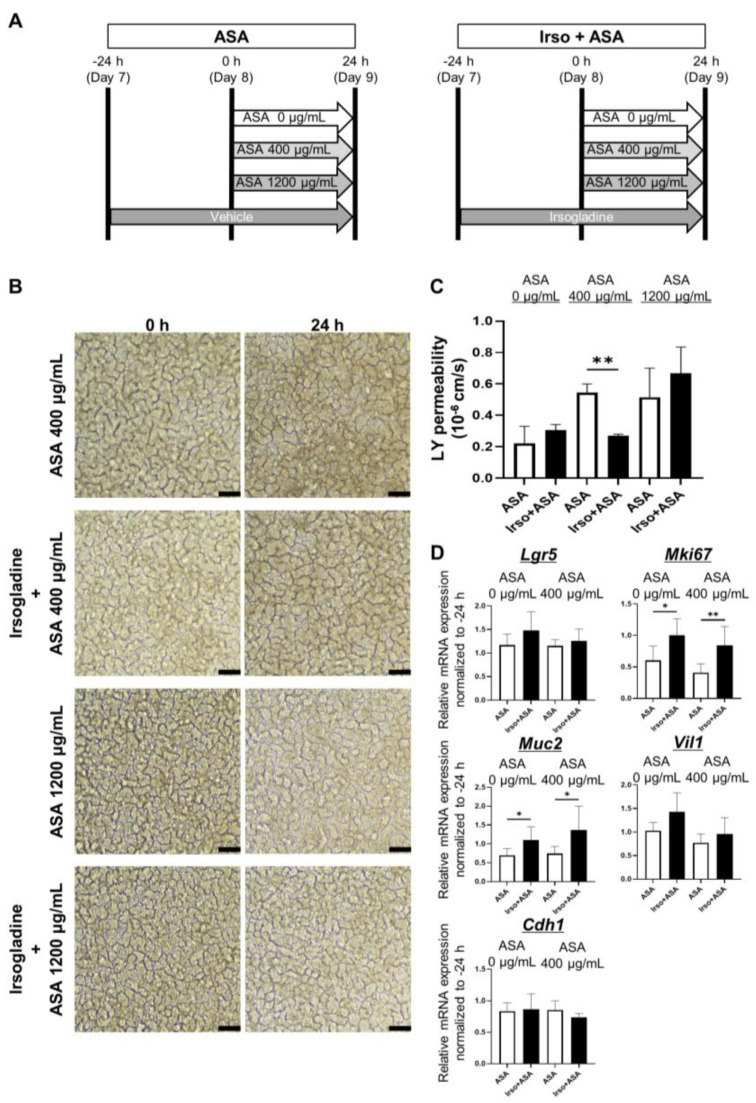
Irsogladine prevented ASA-induced small intestinal injury. (**A**) Protocol for ASA-induced small intestinal injury in 2D-hiPSC-SI. Irsogladine was administered on the basal side of 2D-hiPSC-SI 24 h before ASA addition. Samples were collected 24 h after ASA addition. (**B**) Phase-contrast microscopy. Time-course analysis showed no significant difference in terms of morphological appearance between the ASA groups and Irso + ASA group for both ASA 400 µg/mL and 1200 µg/mL. Scale bars, 100 µm. (**C**) LY permeability of the ASA + Irso group was significantly lower than that of the ASA 400 µg/mL group (*p* < 0.01); mean ± SD; *n* = 3. ASA 400 µg/ml treatment significantly increased the LY permeability compared to ASA 0 µg/ml (*p* < 0.01); mean ± SD; *n* = 3. Statistical analysis was performed using two-tailed Student’s *t*-test; * *p* < 0.05, ** *p* < 0.01. ASA; acetylsalicylic acid, Ctrl: control, Irso; Irsogladine, LY: Lucifer yellow. (**D**) Irsogladine significantly increased *Mki67* and *Muc2* mRNA expression under both normal conditions (ASA 0 µg/mL) and in the presence of 400 µg/mL ASA (*p* < 0.05 and *p* < 0.01; *p* < 0.05 and *p* < 0.05, respectively); mean ± SD; *n* = 6. Statistical analysis was performed using two-tailed Student’s *t*-test; * *p* < 0.05, ** *p* < 0.01. ASA: acetylsalicylic acid.

The effect of Irsogladine against ASA-induced small intestinal injury was investigated by adding Irsogladine to the basal side of 2D-hiPSC-SI and incubating for 24 h before ASA addition (Figure 5A). Samples were collected 24 h after ASA addition. Irsogladine produced no significant macroscopic findings in either the ASA 400 µg/mL or 1200 µg/mL groups (Figure 5B). However, Irsogladine significantly decreased LY permeability in the ASA 400 µg/mL group (*p* < 0.01) (Figure 5C).

Real-time quantitative RT-PCR analysis of cultured 2D-hiPSC-SI revealed that Irsogladine significantly increased *Mki67* and *Muc2* expression under both normal conditions (ASA 0 µg/mL) and in the presence of 400 µg/mL ASA (*p* < 0.05 and *p* < 0.01; *p* < 0.05 and *p* < 0.05, respectively) (Figure 5D).

**Figure 6:**
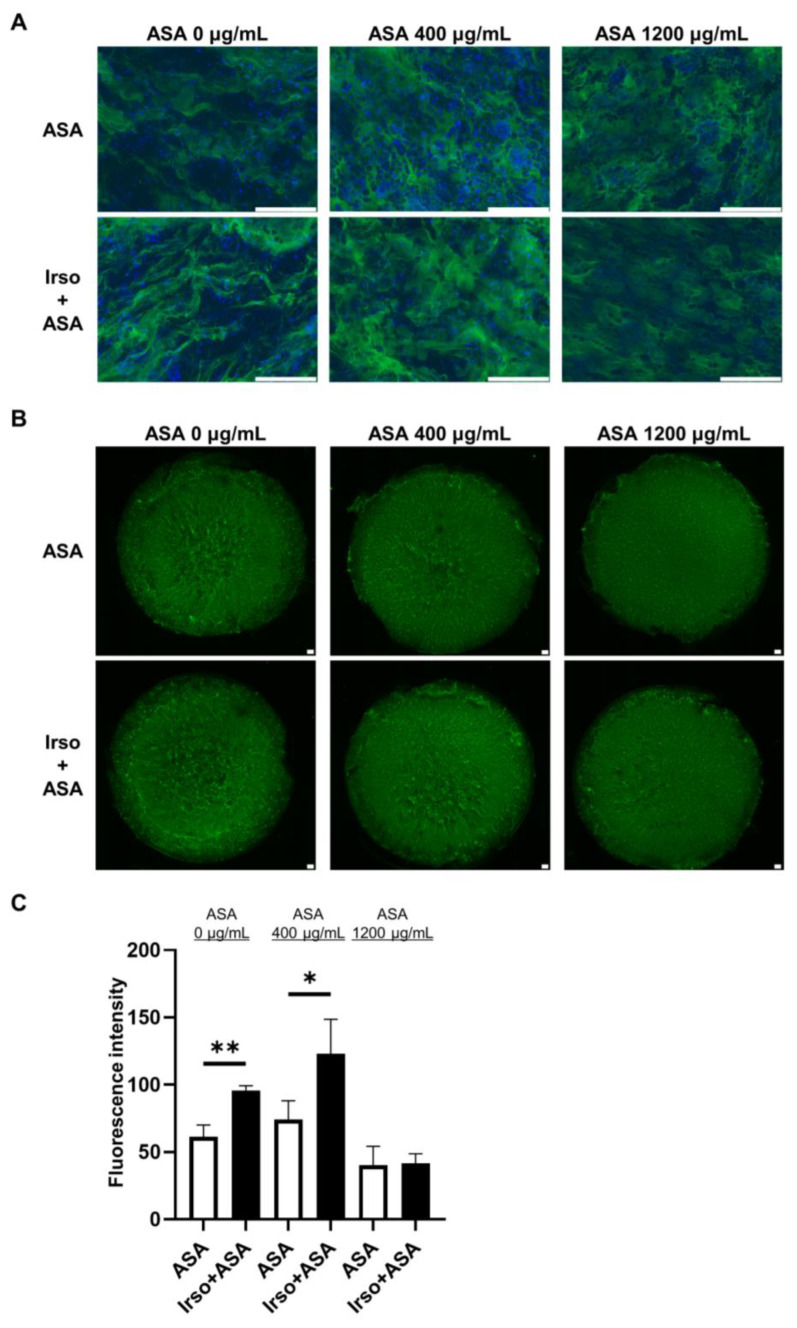
MUC2 expression in ASA-injured 2D-hiPSC-SI after Irsogladine administration. (**A**) Immunofluorescence staining for MUC2 (green). Upper panels, ASA group; lower panels, Irso + ASA group. Nuclei were counterstained with DAPI (blue). Scale bars, 100 µm. (**B**) Representative images of immunofluorescence staining for MUC2 (green) based on fluorescence intensity. Upper panels, ASA group; lower panels, Irso + ASA group. Scale bars, 100 µm. (**C**) Comparison of MUC2 fluorescence intensity between the ASA group and Irso + ASA group. MUC2 fluorescence intensity was calculated in arbitrary units based on threshold color using ImageJ software. Data were obtained from 3 independent sections in each sample. Mean ± SD; *n* = 3. Statistical analysis was performed using two-tailed Student’s *t*-test; * *p* < 0.05, ** *p* < 0.01. ASA; acetylsalicylic acid, Irso; Irsogladine.

MUC2 protein expression in 2D-hiPSC-SI cultured with different ASA concentrations was examined by immunofluorescence staining (Figure 6A). Irsogladine significantly increased the fluorescence intensity of MUC2 under normal conditions (ASA 0 µg/mL) and in the presence of 400 µg/mL ASA (*p* < 0.01 and *p* < 0.05, respectively) (Figure 6B,C).

## 4. Discussion

In this study, we describe a novel 2D-hiPSC-SI experimental model that reconstitutes in vivo human aspirin-induced small intestinal injury. 2D-hiPSC-SI cultured at the ALI showed multi-lineage differentiation of small intestinal epithelium, including absorptive cells, goblet cells, enteroendocrine cells, and Paneth cells, which express CD10, MUC2, chromogranin A, and lysozyme, respectively (Figure 1D). In addition, RNA-ISH revealed ISCs that express *Lgr5* (Figure 1E). Thus, our 2D-hiPSC-SI reconstitutes the turnover of small intestinal epithelial cells derived from iPSCs while maintaining the polarity of the crypt-villus structure.

Many previous reports have described animal models for studying small intestinal mucosal injury associated with NSAIDs, including diclofenac and indomethacin [28,29,30]. However, there have been few reports of animal research models useful for examining LDA-induced small intestinal injury, because it is difficult to induce small intestinal mucosal damage in mice via administration of ASA alone [16,31]. Indeed, endoscopic findings have shown that LDA-induced small intestinal injury is milder than that caused by non-ASA NSAIDs [32]. In addition to the difference between ASA and other NSAIDs, there are differences between animal models and human tissues. The immune system of laboratory mice differs from that of human adults, in that it is more similar to the immature immune system of newborn humans [33]. Moreover, the microbiota of animal models also differs from the human microbiota. Systematic reviews have identified a gap between animal research and human clinical practice, and this may be due to the failure of animal models to adequately mimic clinical disease [34,35]. To overcome these problems, it is necessary to perform experiments using human-derived samples for research into human diseases and the development of drugs for treatment. Here, we developed an ASA-induced small intestinal injury model using 2D-hiPSC-SI and confirmed that the 2D-hiPSC-SI injury is induced in an ASA dose-dependent manner (Figure 2).

Previous studies suggested that non-ASA NSAID-induced small intestinal injury is correlated with increased epithelial permeability [36,37,38]. However, the mechanism of ASA-induced small intestinal injury, including the correlation with small intestinal epithelial permeability, remains unclear due to the lack of adequate experimental models to evaluate the permeability of the human small intestinal epithelium. In this study, we showed that ASA reduces the expression of multiple epithelial cell markers, such as *Muc2* (goblet cell marker), *Vil1* (absorptive cell marker), *Chga* (enteroendocrine cell marker), and *Lyz* (Paneth cell marker), as well as the ISC marker *Lgr5*. These results suggest that ASA-induced small intestinal injury is associated with ISC dysfunction that leads to a reduction in multi-lineage small intestinal epithelial cell differentiation. Furthermore, we showed that ASA increases LY permeability in 2D-hiPSC-SI, which is consistent with previous reports of NSAID-induced small intestinal injury [25,36]. LY permeability was significantly greater in the ASA 400 µg/mL and 1200 µg/mL groups than in the control group at 24 h (*p* < 0.01 and *p* < 0.001, respectively). In ASA 400 µg/mL group, the significant difference was resolved at 48 h after the addition of ASA. Mucosal permeability is attributed to intercellular junctions and thickness of the mucosal layer [39,40]. As shown in IHC staining, 2D-hiPSC-SI had abundant goblet cells. Although ASA 400 µg/mL reduces the expression of multiple epithelial cell markers including *Muc2* (goblet cell marker), the crypt-villus structure of 2D-hiPSC-SI was sustained as shown in Figure 2A. These results suggests that goblet cells were still abundant, even though the number of goblet cells decreased. It has been reported that aspirin administration temporarily increases mucin secretion [41]. Therefore, ASA administration in 2D-hiPSC-SI might induce mucin secretion of goblet cells. We consider that secreted mucin layer decreased mucosal LY permeability at 48 h after 400 µg/mL of ASA administration. The mechanism how ASA affects goblet cell differentiation in small intestine remains to be elucidated. In esophageal cancer cells, ASA reduces COX-2 expression and inhibits NF-κB nuclear migration, which mediates MUC2 overexpression [42,43]. Therefore, it is possible that NF-κB signaling pathway regulates goblet cell differentiation of small intestine post ASA-induced injury.

We also examined the effect of Irsogladine on normal small intestine and ASA-induced small intestinal injury (Figure 5). Under normal conditions without ASA, Irsogladine significantly increased *Mki67* and *Muc2* expression in 2D-hiPSC-SI. These results suggest that Irsogladine promotes turnover and goblet cell differentiation in the small intestinal epithelium. Although Irsogladine did not affect small intestinal mucosal permeability under normal conditions, Irsogladine suppressed the increased mucosal permeability induced by ASA (400 µg/mL) in the model of ASA-induced small intestinal injury. Irsogladine also increased *Mki67* and *Muc2* expression, which had been reduced in ASA-induced small intestinal injury. The small intestinal epithelium is coated by a secreted mucus layer. MUC2 expressed by goblet cells is a major component of the gel-like secreted mucus layer that coats small intestinal epithelium [44,45,46]. This gel-like mucus layer is associated with mucosal permeability and plays an important role in intestinal barrier function. Therefore, goblet cell dysfunction can lead to increased mucosal permeability, which can in turn lead to bacterial invasion or disorders of the immunity system that are associated with several intestinal diseases, such as inflammatory bowel disease. Here, we showed that Irsogladine plays a protective role in maintaining small intestinal mucosal permeability against ASA-induced injury by promoting goblet cell differentiation. Irsogladine is thus a candidate protective drug for ASA-induced small intestinal injury as well as leaky gut syndrome or functional dyspepsia, which are associated with increased intestinal permeability [47,48,49].

There are some limitations in this study. First, our 2D-hiPSC-SI model does not include immune cells or microbiota. Microbiota organisms can be added to the apical surface of the 2D-hiPSC-SI, and immune cells can be co-cultured within the basal medium. In the future, we will conduct in vitro experiments to reconstitute the whole in vivo environment, including immune cells and microbiota. Second, the effect of Irsogladine in vivo remains unclear. Prospective and randomized controlled studies are necessary to elucidate the preventative effect of Irsogladine in ASA-induced small intestinal injury.

## 5. Conclusions

In conclusion, we have established a novel ASA-induced small intestinal injury model using human iPSC-derived small intestine. Irsogladine maintains mucosal permeability and goblet cell differentiation against ASA-induced small intestinal injury.

## Data Availability

The data presented in this study are available on request from the corresponding author (Takahito Katano).

## Supplementary Materials

The following supporting information can be downloaded at: https://www.mdpi.com/article/10.3390/medicina59010092/s1, Figure S1. Culture protocol for human iPSC-derived 2D monolayer crypt-villus structural small intestine (2D-hiPSC-SI). Figure S2. Claudin-7 expression in ASA-injured 2D-hiPSC-SI after Irsogladine administration. Table S1. Sequences of primers used for real-time PCR analysis.
