# Supplementary material for: Protective Effect of Irsogladine against Aspirin-Induced Mucosal Injury in Human Induced Pluripotent Stem Cell-Derived Small Intestine
Source: Medicina (Kaunas). 2022 Dec 31;59(1):92. doi: 10.3390/medicina59010092 (PMC9863323; doi:10.3390/medicina59010092)
Supplement: Supplementary file 1 [file medicina-59-00092-s001.zip › Figre S2_R1_katano.pdf]

Figure S2.

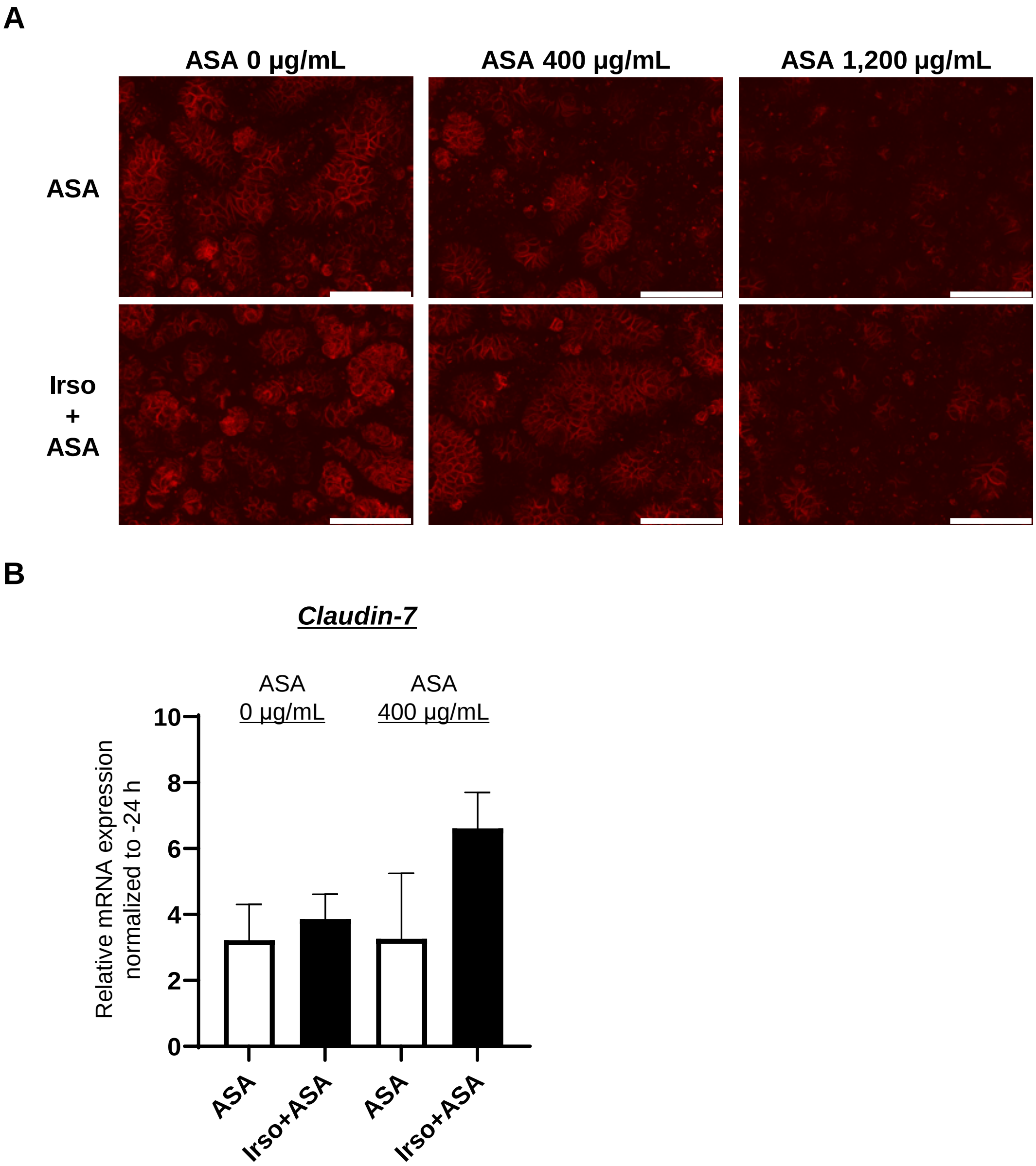

Figure S2. Claudin-7 expression in ASA-injured 2D-hiPSC-SI after Irsogladine administration.
(**A**) Immunofluorescence staining for Claudin-7 (red). Upper panels, ASA group; lower panels, Irso + ASA group. Scale bars, 100 µm. (**B**) Irsogladine tended to increase Claudin-7 mRNA expression in the presence of 400 µg/mL ASA, but the difference was not statistically significant; mean $\pm$ SD; n = 3.
