# Supplementary material for: Protective Effect of Irsogladine against Aspirin-Induced Mucosal Injury in Human Induced Pluripotent Stem Cell-Derived Small Intestine
Source: Medicina (Kaunas). 2022 Dec 31;59(1):92. doi: 10.3390/medicina59010092 (PMC9863323; doi:10.3390/medicina59010092)
Supplement: Supplementary file 1 [file medicina-59-00092-s001.zip › Figure S1.pdf]

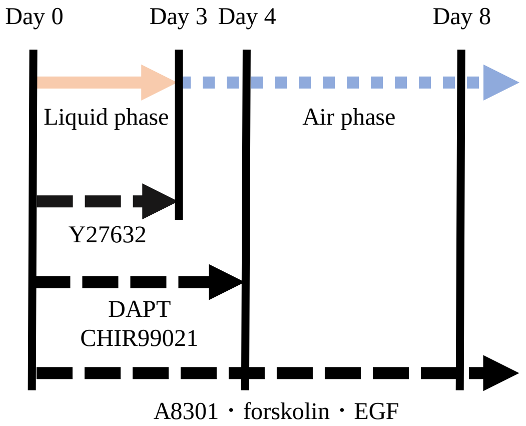

Figure S1. Culture protocol for human iPSC-derived 2D monolayer crypt-villus structural small intestine (2D-hiPSC-SI).
Cells were seeded from human iPSC-derived small intestinal budding organoids and cultured at the air-liquid interface. In the first 3 days, 10 μM Y-27632, 2.5 μM DAPT, 3 μM CHIR99021, 500 nM A8301, 10 μM forskolin, and 100 ng/mL EGF were added to conditioned medium. On day 3, the medium was changed to conditioned medium supplemented with 2.5 μM DAPT, 3 μM CHIR99021, 500 nM A8301, 10 μM forskolin, and 100 ng/mL EGF. On day 4, the medium was changed to conditioned medium supplemented with 500 nM A8301, 10 μM forskolin, and 100 ng/mL EGF.
