# Supplementary material for: Protective Effect of Irsogladine against Aspirin-Induced Mucosal Injury in Human Induced Pluripotent Stem Cell-Derived Small Intestine
Source: Medicina (Kaunas). 2022 Dec 31;59(1):92. doi: 10.3390/medicina59010092 (PMC9863323; doi:10.3390/medicina59010092)
Supplement: Supplementary file 1 [file medicina-59-00092-s001.zip › Table S1_Medicina_R1.pdf]

Gene name	Sense (5'→3')	Antisense (5'→3')
Chga	TCCGACACACTTTCCAAGCC	TTCTGCTGATGTGCCCTCTC
Cdh1	ATGAGTGTCCCCCGGTATCT	GGTCAGTATCAGCCGCTTTC
Hprt	CTTTGCTTTCCTTGGTCAGG	TCAAGGGCATATCCTACAACA
Mki67	GACTTTGGGTGCGACTTGAC	ACCCCGCTCCTTTTGATAGT
Lgr5	TGCTCTTCACCAACTGCATC	CTCAGGCTCACCAGATCCTC
Lyz	TCAATAGCCGCTACTGGTGT	AATGCCTTGTGGATCACGGA
Muc2	AGAAGGCACCGTATATGACGAC	CAGCGTTACAGACACACTGCTC
Vil1	AGCCAGATCACTGCTGAGGT	TGGACAGGTGTTCCTCCTTC
